# Understanding the Transvalvular Gradient in Aortic Stenosis: A Multifaceted Perspective

**DOI:** 10.3390/jcm14227916

**Published:** 2025-11-07

**Authors:** Giovanni La Canna, Sara Habjan, Iside Scarfò

**Affiliations:** 1 Applied Diagnostic Echocardiography Unit, IRCCS Humanitas Clinical and Research Hospital, 20089 Rozzano, Italy; isidestella.scarfo@gmail.com; 2 Cardiology Department, University Medical Center Ljibijana, 1000 Ljubljana, Slovenia; sara.hbjnl@gmail.com

**Keywords:** aortic stenosis, transvalvular gradient, aortic valve reserve

## Abstract

Growing age-related epidemiology, together with an increasing burden of cardiac co-pathology and comorbidities, has progressively subverted the clinical paradigm of Aortic Stenosis (AS) towards a multifaceted scenario. Timely surgical or transcatheter valve replacement is paramount to reduce morbidity and mortality in AS patients provided that the obstruction is hemodynamically important and responsible for the symptoms across a variety of clinical contexts. Despite its recognized role in AS assessment severity, transvalvular gradient (TVG) reflects complex interplay among anatomical, mechanical and fluid-dynamic factors, challenging the ultimate recognition of significant aortic valve obstruction. Careful phenotyping of TVG by assessing its underlying variables may enhance diagnostic work-up, risk stratification and management of AS. Emerging imaging modalities, such as three-dimensional echocardiography, automatic flow and myocardial function assessment, and advanced fluid dynamics analysis are promising for refining multifaceted TVG phenotypes. A deeper understanding of the substrate underlying TVG may add new insight into the trajectory of valve obstruction and its interaction with left ventricular function, thereby supporting the tailoring of TVG-guided clinical strategies of the evolving scenario of AS.

## 1. Introduction

Aortic stenosis (AS) is a growing age-related valvular disease with a high cardiac co-pathology and comorbidity burden [1,2,3,4]. Timely surgical or transcatheter valve replacement is paramount to reduce mortality and morbidity in AS patients, provided the obstruction is hemodynamically significant and directly responsible for the symptoms [5]. Transvalvular gradient (TVG), invasively measured by catheterization or non-invasively by Doppler echocardiography, is the most commonly used parameter for AS diagnosis [6]. Despite its recognized role in AS assessment, TVG reflects a complex interplay of anatomical, mechanical, and fluid-dynamic factors, challenging the ultimate TVG-guided recognition of significant valve obstruction [7]. Although higher TVG values suggest more severe AS, a clear relationship between TVG and symptoms occurrence is not always observed [8]. Symptomatic and asymptomatic patients frequently exhibit overlapping TVG. In addition, the clinical scenario of AS may include varying phenotypes, with a heterogeneous combination between severity of aortic valve obstruction and TVG entity, necessitating a nuanced approach to assessment [9].

To address inconsistencies in TVG-based assessment, an integrated evaluation framework considers flow state and left ventricular function. Low TVG may occur in low-flow conditions, with or without overt left ventricular dysfunction, requiring additional parameters, such as valve area or re-testing following flow normalization, to achieve the ultimate recognition of severe valve obstruction [10,11]. However, AS progression, together with flow conditions and LV function changes, may determine progressive or regressive (declining) TVG trajectories, leading to a different clinical course and outcome. Understanding the underlying factors influencing TVG may provide new insight into to the interplay between the trajectory of valve obstruction and left ventricular function, facilitating tailored clinical strategies. Finally, the inconsistency between TVG severity and symptoms is an intriguing issue in the setting of severe AS and comorbidity to explain the true etiology of clinical symptomatic burden and guide appropriate treatment.

Phenotyping TVG according to underlying variables may enhance the diagnostic work-up, risk stratification, and management of AS. Emerging imaging modalities, including three-dimensional echocardiography, cardiac magnetic resonance, automated flow and myocardial function assessment, and fluid dynamics analysis, offer promising pathways to further refine TVG phenotypes.

This perspective focuses on the evolving role of TVG in the diagnosis, prognosis, and management of AS. By capturing the multifaceted nature of TVG, clinicians can more effectively address the varying clinical scenarios associated with AS.

## 2. Transvalvular Aortic Gradient: A Multifaceted Parameter of Aortic Stenosis

TVG is a well-established diagnostic marker that is central to assessing the hemodynamic burden of AS. Doppler echocardiography, using the Bernoulli equation [12], is recognized as the standard estimation of TVG. A mean TVG ≥ 40 mmHg is the established threshold for severe AS, but measurement is influenced by multiple factors, limiting its reliability as an absolute parameter for AS severity [11,13,14]. Extremely high gradient (peak > 100 mm Hg) or peak velocity greater than 5.5 m/sec indicate advanced AS and portend an unfavorable clinical course [15]. Otherwise, TVG requires comprehensive assessment to determine the true severity of AS [11,13]. The Aortic Valve Area (AVA), derived from the Doppler Continuity Equation or the invasive Gorlin formula, provides a quantitative measure of valve narrowing. Direct planimetry with 2D or 3D echocardiography or CT further refines this assessment [16,17]. An AVA < 1.0 cm^2^ is considered indicative of significant AS, though indexing to body surface area is essential in small patients. The concordance of a mean TVG ≥40 mm Hg with an AVA < 1 cm^2^ usually confirms severe AS; nevertheless, discordant patterns are frequent and represent a major diagnostic challenge [18].

### 2.1. Transvalvular Aortic Gradient and Pressure Recovery

Due to the reduction in AVA, an increase in blood velocity is required to maintain stroke volume (SV) across the obstructed valve. The rise in blood velocity across the aortic valve occurs at the expense of left ventricular pressure. The site where the transformation of pressure energy into blood flow velocity is maximized is called the *vena contracta*. At this point, the measurement of transvalvular pressure loss coincides with the maximum velocity increase. Beyond the *vena contracta*, part of the kinetic energy is dissipated as turbulence and heat, while a portion is reconverted into pressure energy (a phenomenon known as *pressure recovery*). Recovered pressure, measurable as wall pressure, contributes to cardiovascular efficiency. Pressure recovery should be considered an advantageous phenomenon, as it allows for a reduced energetic cost under improved rheological conditions for the same TVG, ultimately decreasing the energy expenditure required for the left ventricle’s propulsive function. The magnitude of pressure recovery depends on ascending aorta dimension, valve orifice shape (e.g., bicuspid aortic valves, asymmetrical stenotic orifice), flow eccentricity, degree of valve orifice narrowing, cardiac output. Failure to account for *pressure recovery* may lead to over-estimation of the TVG, with moderate AS being misclassified as severe. Conversely, catheter-based measurements taken distal to the *vena contracta* may underestimate TVG. *Pressure recovery* is most pronounced with small ascending aorta, asymmetrical or elongated stenotic orifice, concentric (non-eccentric) aortic valve flow, high-flow states, non-severe AS. Because pressure recovery reduces the energetic cost for a given TVG, it can be quantified using the *energy loss index* (ELI, cm^2^/m^2^) proposed by Garcia and Pibarot [19,20,21]. The formula incorporates the effective orifice area (EOA), ascending aortic cross-sectional area (AA), and body surface area (BSA).

$$ELI=\frac{EOA\times AA}{AA-EOA}\;÷BSA$$

where EOA = Effective orifice area (calculated via the continuity equation); AA = Ascending aortic cross-sectional area 1 cm above the valve plane; BSA = Body surface area. The aortic cross-sectional area (AA)

$$AA=\pi \times {\left(\frac{D_{A}}{2}\right)}^{2}$$

where D_A_ is the aortic diameter (cm).

**Figure 1:**
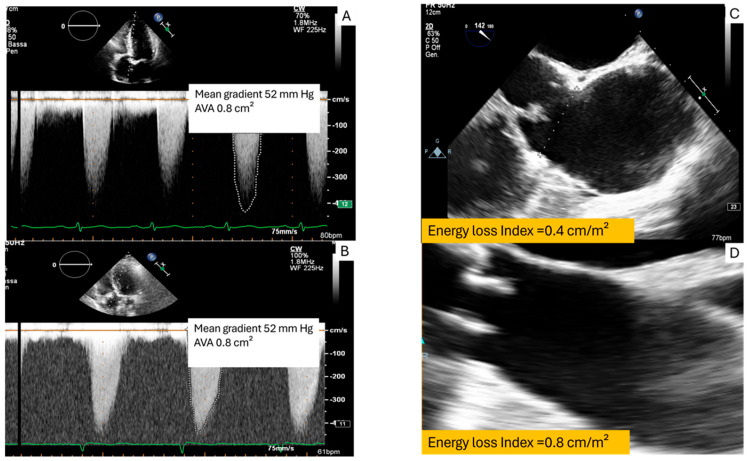
Echocardiographic assessment of severe aortic stenosis with a mean gradient of 52 mmHg and an AVA of 0.8 cm^2^. Depending on whether the ascending aorta was enlarged (**C**) or normal in size (**D**), the energy loss index (ELI) ranged from 0.4 to 0.8 cm^2^/m^2^, underscoring its role in refining severity classification beyond conventional Doppler indices. (**A**,**B**): Doppler transthoracic echocardiography in two different patients with the same mean gradient; (**C**,**D**): transesophageal echocardiography with different ascending aortic size.

At the same TVG, a higher hemodynamic burden can be anticipated in cases of ascending aorta dilation, an eccentric flow jet, or a cup-shaped stenotic geometry [22,23]. Figure 1 shows two patients with different ELI due to ascending aorta dimensions, despite having the same high TVG. Significantly, *pressure recovery* may cause echocardiography to overestimate TVG, since it does not account for energy regained downstream. In cases where pressure recovery is substantial, TVG may appear high, but ELI could still be in the range of non-severe obstruction, indicating a lower energetic cost and a more favorable prognosis [24]. Studies suggest that ELI correlates more closely with symptomatic status and long-term prognosis than EOA alone [24]. An ELI < 0.60 cm^2^/m^2^ indicates true severe AS, whereas a low EOA with a preserved ELI may indicate significant pressure recovery and potential overestimation of AS severity if ELI is not considered. Clinically, patients with EOA < 1 cm^2^ but ELI > 0.60 cm^2^/m^2^ may have mild to moderate AS and could safely defer valve replacement despite a significant TVG. Conversely, ELI < 0.60 cm^2^/m^2^ confirms severe AS, reinforcing the indication for surgical or transcatheter aortic valve replacement (SAVR/TAVR). Accurate ELI calculation requires careful aortic diameter and LVOT measurement. Although not yet included in current guidelines, ELI provides a physiologically refined index that avoids misclassification, unnecessary interventions, and better aligns with functional status and prognosis. Its integration into echocardiographic assessment, particularly in small aortic roots or discordant grading, may enhance clinical decision making.

### 2.2. Flow and Transvalvular Aortic Gradient Relationship

The relationship between flow (volume passing through the valve per unit time) and TVG (pressure or velocity difference across the valve) is governed by fundamental hemodynamic principles, primarily derived from the Bernoulli equation. According to the simplified Bernoulli equation, which ignores viscous losses and the effects of flow acceleration, TVG is derived with a formula ΔP = 4⋅v2 (where P is derived pressure and v is the velocity of blood flow). Since flow (Q) is related to velocity (v) and cross-sectional area (A) by: Q = A⋅v, TVG is approximately proportional to flow squared, meaning that an increase in flow leads to a disproportionate increase in pressure gradient. Indeed, flow and TVG have a non-linear (quadratic) relationship. In clinical terms, high-output states can exaggerate pressure gradients across stenotic valves, whereas low-flow states can mask the severity of stenosis, leading to underestimation of disease severity unless flow is normalized. Consequently, in the presence of fixed AS, we can observe a varying spectrum of TVG according to flow state. Even though it is a key indicator of cardiac function, EF alone may be unable to categorize transvalvular flow subtending TVG. Indeed, SV is an absolute volume (mL/beat), while EF is a percentage (%) of EDV. SV measures how much blood the heart pumps per beat (absolute volume ml/beat), whereas EF measures how efficiently the ventricle ejects blood (as a percentage of end-diastolic volume). SV can be normal even when EF is low (e.g., in conditions with increased end-diastolic volume, such as dilated cardiomyopathy). On the other hand, reduced SV may occur despite normal EF in the presence of a small hypertrophic left ventricle. Thus, TVG in aortic stenosis may be categorized according to flow state and EF in clinically relevant subtypes, such as normal or high-flow high gradient, low-flow low gradient, with or without EF reduction.

#### 2.2.1. Phenotyping Flow Conditions in Aortic Stenosis

Phenotyping flow conditions underlying AS is crucial to better understand the disease and tailor its management. Several studies report that patients with a significant mean TVG (≥40 mm Hg) experience adverse clinical outcomes when their SV, adjusted for body mass index (SVi), is less than 35 mL/m^2^. Lancellotti et al. conducted a prospective study re-classifying the TVG, which defines severe AS based on a mean TVG above or below 40 mmHg, into subgroups according to normal (≥35 mL/m^2^) or reduced (<35 mL/m^2^) SV [25]. This study underscores the importance of incorporating SVi into the evaluation of patients with AS to unmask low flow despite a preserved ejection fraction. Rusinaru et al. reported a poor prognosis in patients with AS and SVi < 30 mL/m^2^ or absolute SV < 55 mL, despite a normal ejection fraction (EF) [26]. Accordingly, patients with similar TVG may exhibit differing hemodynamics and outcomes, depending on flow conditions estimated using SV. Based on SV, it is possible to identify distinct profiles of TVG, leading to a related nomenclature (low-flow, normal-flow, or high-flow high TVG) that may enhance the prognostic categorization of AS. However, Lønnebakken et al. [27] found that SVI < 35 mL/m^2^ was associated with a 47% increased risk of major CV events in multivariable analysis (HR 1.47, 95% CI 1.16 to 1.87, *p* = 0.001), while no significant association with all-cause mortality was found. Additionally, a relatively recent study suggests the SVi threshold predicting adverse outcomes after aortic valve replacement is sex-specific, with values of <32 mL/m^2^ for women and <40 mL/m^2^ for men [28]. Although low SV should be regarded as a marker of increased risk, inconsistency remains in its respective cut-off and sex-related differences on SVi values for defining low flow in AS [29].

##### Challenges in SV Estimation

SV estimation in patients with AS can be challenging due to several factors that may affect the accuracy and reliability of the measurements. Doppler-derived SV is based on the conventional formula

$${LVOT}_{area}=\pi \times {\left(LVOT\;diameters/2\right)}^{2}$$

Small inaccuracies in LVOT diameter measurement can significantly affect the calculated aortic valve area due to the squared term in the continuity equation. Furthermore, the LVOT is often assumed to be circular, whereas it may be elliptical, leading to underestimation or overestimation of SV [30]. Additionally, errors in flow measurement at the LVOT, such as inaccuracies in the velocity-time integral caused by a poor acoustic window or a misaligned Doppler angle, can further compromise the accuracy of stroke volume estimation. Dynamic LVOT changes in some conditions (e.g., hypertrophic cardiomyopathy) may introduce an additional source of error. An alternative approach uses volumetric (3D or Biplane) method. Stroke Volume SV = LVEDV − LVESV, LVEDV = Left ventricular end-diastolic volume LVESV = Left ventricular end-systolic volume, measured using the biplane Simpson’s method (2D echocardiography) or 3D echocardiography. Each approach has pitfalls that should be carefully considered.

#### 2.2.2. Influence of Hemodynamic Factors

Changes in preload (e.g., dehydration or diuretics) or afterload (e.g., systemic arterial hypertension) may alter SV without necessarily reflecting the severity of AS. Therefore, careful attention should be given to identifying and correcting underlying load conditions, particularly systemic arterial hypertension and low-preload conditions, to avoid misleading estimates of SV in patients with AS [31].

#### 2.2.3. Comorbid Cardiac Conditions

Primary LV dysfunction or concomitant valve disease can impair anterograde forward aortic flow independently of AS severity. Atrial fibrillation can result in beat-to-beat variation, making accurate estimation challenging. Dynamic flow conditions may be a source of high-output states (e.g., anemia, fever).

#### 2.2.4. Refining Flow State: Stroke Volume vs. Flow Rate

SV, despite its potential limitations, remains a robust parameter to assess flow state and the hemodynamic burden of AS. Indexed SV (Svi) is a critical variable to estimate AVA using the Continuity Equation (CE). AVA-CE values < 1 cm^2^ (or <0.6 cm^2^/m^2^) are commonly used cutoffs for AS severity. However, SV (ml/beat) does not directly quantify flow, unlike the Gorlin formula, which incorporates flow rate (FR:CO/SEP) to calculate AVA. FR by including systolic ejection period, and thus may better reflect valve resistance, as well as being less influenced by body size offering potential advantages in obese patients (low SVi but normal FR) and small women (normal SVi but low FR) [32,33]. However, clinical studies are conflicting. In the SEAS study, Saeed et al. reported that low FR (<200 mL/s) independently predicted CV and all-cause mortality in asymptomatic, non-severe AS [33]. Similarly, Vamvadikou et al. found low FR predictive of risk in symptomatic patients undergoing AVR, even with preserved EF [34]. Yet prognostic consistency remains limited, partly due to lack of HR correction. As Bache et al. noted, the relationship between SV, HR, AVA, and ejection time is weak; a wide range of ejection times for a given AVA may dissociate SV and FR [35]. Sen et al. observed 62% concordance between low SVi (<35 mL/m^2^) and low FR (<200 mL/s) in 621 patients with severe AS (indexed AVA < 0.6 cm^2^/m^2^). Classical low-flow AS (low SVi and low FR) carried the worst prognosis, but FR did not significantly improve risk stratification. Importantly, discrepancies were largely HR-driven: patients with low SVi/normal FR had higher HR (~85 bpm) and shorter LVET, whereas those with normal SVi/low FR had lower HR (~65 bpm) and longer LVET [36]. Overall, SV remains the most widely used index of flow, but FR, by incorporating ejection time, may provide a more accurate assessment. Nonetheless, variability in HR and body size demands an integrated evaluation to avoid misclassification when SV and FR diverge [36,37,38,39,40].

## 3. Flow-Related Transvalvular Aortic Gradient/Aortic Valve Area Matching

Matching flow and TVG with AVA is essential to accurately classify the severity and prognosis of AS. By incorporating SVi and/or FR, TVG can be contextualized to refine the assessment of true hemodynamic obstruction and reduce flow-related misinterpretations. Depending on the flow state, TVG may reflect high, normal, or low flow conditions. Because of its flow dependence, AVA derived from the continuity equation is usually interpreted in conjunction with TVG when evaluating AS severity. Nonetheless, a substantial proportion of cases show discrepancies between AVA and TVG, resulting in discordant grading. Such discordance complicates both the recognition of true AS severity and its clinical management.

### 3.1. High Transvalvular Gradient with Discordant Aortic Valve Area (>1 cm^2^)

Elevated TVG may occur without severe anatomic stenosis when transvalvular flow is increased. High-flow states such as severe anemia, sepsis, hyperthyroidism, pregnancy, or arteriovenous fistula, as well as exercise, tachyarrhythmias, inotropes, or pre-dialytic conditions, can transiently generate a high-gradient profile. Recognition of these reversible states is crucial to avoid misclassification of “false extreme” AS. Discordance may also arise from mismatch between stroke volume (SV) and flow rate (FR), e.g., reduced SVi in large body surface area or increased FR due to tachycardia. Approximately 5–7% of AS patients present with high gradients (mean TVG > 40 mmHg) despite AVA > 1 cm^2^ [41,42,43], often with bicuspid valves. Pressure recovery in elongated stenoses and technical errors in continuity-equation–derived AVA (e.g., LVOT diameter mismeasurement, Doppler misalignment) may further explain this discordance. Post-extrasystolic beats can similarly increase TVG despite preserved AVA through augmented contractility and reduced valvular resistance.

Role of flow rate. FR, by integrating SV and ejection time, better reflects hemodynamics than SV alone. An AVA > 1 cm^2^ with FR > 300 mL/s suggests a flow-driven rather than truly severe gradient. Incorporation of FR alongside AVA, mean gradient, and SVi improves classification, especially in patients with normal SVi but elevated heart rate. Volumetric SV assessment, less affected by LVOT geometry, may refine grading further.

Imaging refinement. In patients with high-gradient AS but AVA > 1 cm^2^, TEE offers superior anatomical and hemodynamic assessment compared with transthoracic echocardiography. TEE enables more accurate LVOT diameter measurement, direct 3D quantification, and reduces sources of error in SV and AVA calculation. Three-dimensional TEE permits direct planimetry of the aortic valve orifice in systole, avoiding the inherent limitations of the continuity equation [44,45]. This approach is relatively independent of flow conditions, making it particularly reliable in high-output states. If direct planimetry yields an AVA < 1 cm^2^, the diagnosis of severe AS is confirmed, even when TTE suggests otherwise. TEE additionally provides detailed evaluation of valve morphology and calcification; severe calcification and restricted leaflet motion strongly support the diagnosis of true severe AS. Notably, patients with a high gradient and AVA > 1 cm^2^ more commonly have bicuspid valves and a less favorable clinical course, with worse outcomes after aortic valve replacement compared with the broader AS population [42].

**Figure 2:**
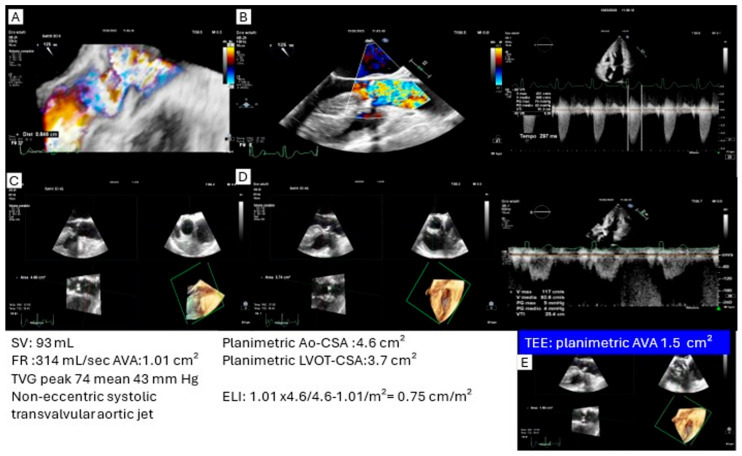
Case of an asymptomatic patient with high transvalvular gradients and discordant AVA (>1 cm^2^). TEE planimetry demonstrated an AVA of 1.5 cm^2^, while the calculated ELI (using direct measurements of aortic and LVOT CSA) was 0.75 cm^2^/m^2^, consistent with functionally non-severe AS. The high gradients were related to a high-flow state with marked pressure recovery due to a small ascending aorta and a non-eccentric transvalvular jet. ((**A**–**E**) transesophageal images of transvalvular aortic jet (**A**): 3d color flow mapping; (**B**): 2d color flow mapping), cross-sectional area left ventricular outflow tract (**C**) and cross-sectional aortic area at sino-tubular junction.

Energy loss index and pressure recovery. In discordant cases with high TVG but AVA > 1 cm^2^, reevaluation of the energy cost associated with the gradient is crucial. Doppler-derived TVG reflects the instantaneous pressure drop across the aortic valve but does not account for downstream pressure recovery, particularly relevant in patients with a small ascending aorta. In such cases, gradients may appear disproportionately elevated relative to the anatomical severity of stenosis. The energy loss index (ELI), which incorporates both AVA and ascending aortic cross-sectional area, provides a more physiologically accurate measurement of valvular obstruction. TEE facilitates ELI calculation by enabling direct measurement of both LVOT and aortic cross-sectional areas, while also allowing detailed assessment of valve morphology and jet eccentricity, thereby refining estimation of pressure recovery in discordant cases. (Figure 2).

### 3.2. High Transvalvular Gradient with Concordant Aortic Valve (<1 cm^2^): What Is the Ultimate Degree of Aortic Stenosis?

The concordance of a high TVG with an AVA < 1.0 cm^2^ (calculated via the continuity equation) is widely accepted as definitive evidence of severe AS. Within this concordant pattern, it is essential to distinguish true extreme AS, characterized by a very high peak TVG (>100 mmHg, peak velocity > 5.5 m/s) [15] and associated with a poor prognosis, from pseudo-extreme AS driven by excessive flow conditions. Accurate correction for high flow when measuring TVG is therefore critical for the definitive phenotyping of extreme AS. Conversely, identifying an underlying low-flow state in the context of a high TVG is equally important, as it carries significant adverse prognostic implications.

### 3.3. Low-Gradient (Mean Gradient < 40 mm Hg) with Discordant Aortic Valve Area (<1 cm^2^)

This phenotype is defined by an AVA < 1.0 cm^2^, an indexed AVA <0.6 cm^2^/m^2^, and a mean TVG < 40 mmHg, with *preserved or reduced* LVEF [46].

#### 3.3.1. A- Low-Flow Low-Gradient, Normal Ejection Fraction

**Figure 3:**
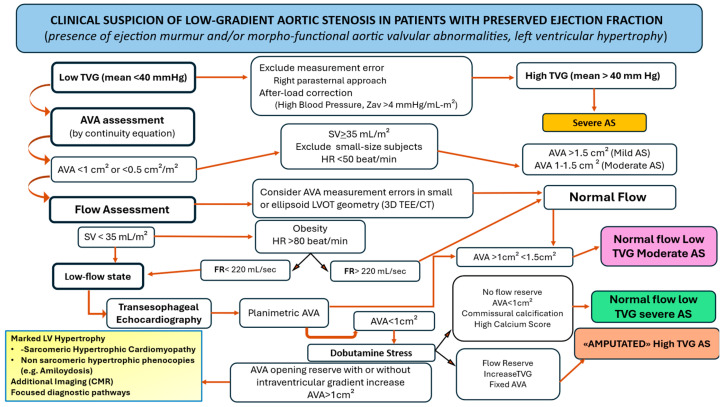
Diagnostic Algorithm for Low-Gradient Aortic Stenosis with Preserved Ejection Fraction.

When LVEF is preserved this phenotype is referred to also as *paradoxical low-flow, low-gradient AS* [47,48,49,50,51,52,53]. The most common substrate of reduced effective anterograde LV flow despite preserved EF is pronounced concentric LV hypertrophy, characterized by a small cavity, impaired diastolic filling, and diminished systolic longitudinal function. This phenotype is commonly associated with systemic hypertension, which may further contribute to the attenuation of TVG [51]. Atrial fibrillation is another common and significant contributor to reduced LV outflow in AS patients with preserved LVEF. Other conditions that may further decrease effective forward LV flow include significant mitral regurgitation, mitral stenosis, tricuspid regurgitation, and right ventricular dysfunction. The diagnostic work-up for *paradoxical low-flow, low-gradient AS* is complex and requires a carefully integrated approach [54,55,56,57] (Figure 3).

*Step1-Verification of measurements.* The accuracy of AVA, TVG, and LVEF measurements and calculations must be confirmed. TVG assessment should be performed from multiple transthoracic echocardiographic windows, including the right parasternal view, to ensure the maximal gradient is captured [58] (Figure 3). Importantly, use of the right parasternal may lead to reclassification of many patients initially diagnosed with paradoxical *low-flow low- gradient AS* into the *high-gradient severe* AS category. Detecting and managing high blood pressure is also crucial, as increased LV impedance can attenuate the TVG. Many patients with paradoxical LFLG AS have elevated valvulo-arterial impedance (Zva), which reflects the combined valvular and arterial components on LV [31,59]. Zva is calculated using the following formula and expressed in mmHg/mL/m^2^: Zva = (mean aortic pressure gradient + systolic brachial artery pressure)/stroke volume index. If systolic blood pressure exceeds 150 mmHg, reassessment of AS severity after optimized antihypertensive therapy is paramount. Zva values > 4.5 mmHg/mL/m^2^ may indicate significant AS even when arterial hypertension is well controlled, whereas values < 4.5 mmHg/mL/m^2^ make severe AS less likely. The interpretation of Zva should also account for corrections related to factors influencing pressure recovery, particularly ascending aortic dimensions, to ensure accurate assessment of valvular obstruction and LV afterload.

*Step 2-Assessment of AVA*. AVA should be measured using the continuity equation. In patients where mean TVG is definitively <40 mmHg, the AVA derived from the continuity equation may guide further classification of AS (mild, moderate, or severe when <1 cm^2^, the latter necessitating careful flow assessment). Potential measurement errors must be excluded, particularly in small or ellipsoid LVOT, by optimizing imaging windows or incorporating data from 3DTEE or CT.

*Step 3-Flow evaluation*. Both TVG and AVA are flow-dependent. In patients with AVA <1 cm^2^, documentation of a low-flow state (stroke volume index < 35 mL/m^2^) is pivotal in explaining the reduced TVG despite preserved LVEF. Since ejection duration may influence effective transvalvular flow, flow rate (FR < 220 mL/sec) can further refine hemodynamic assessment, particularly in patients whose heart rate or body surface area falls outside the physiological range and thus compromises the SVi reliability. In small or eccentric LVOT [60,61], direct measurement of the LVOT cross-sectional area using 3D TEE or CT enables flow accuracy and allows recalculation of AVA [30,44]. Beyond accurate LVOT measurement, 3D TEE or CT provide direct planimetry of the AVA and assessment of leaflet calcification. A planimetric AVA < 1 cm^2^, commissural calcification, and high calcium score (>2000 AU in men, >1500 AU in women) support the diagnosis of severe LFLG AS.

*Step 4-Dobutamine stress echocardiography*. Dobutamine stress echocardiography is pivotal when normalization of LV flow can distinguish true severe AS—confirmed by fixed planimetric AVA and unmasked gradient—from pseudo-severe AS, in which valve opening increases to >1 cm^2^. In patients showing increased valve opening under dobutamine, alternative diagnoses should be considered, particularly marked myocardial hypertrophy due to sarcomeric cardiomyopathy or hypertrophic phenocopies, which require specific diagnostic pathways. Notably, 10–20% of patients with paradoxical LFLG AS have concomitant wild-type transthyretin (ATTR) amyloidosis, leading to pronounced LV hypertrophy, a small LV cavity, restrictive filling, and low SVi despite preserved EF [62]. Recognition is critical given its major prognostic and therapeutic implications.

In conclusion, the diagnosis of low-gradient, low-flow AS with preserved ejection fraction may be acceptable if the greatest peak gradient is accurately measured, true flow is properly assessed (e.g., LVOT area by 3D echocardiography including flow rate measurement), the anatomic valve area is confirmed to be less than 1 cm^2^ (at baseline and/or during dobutamine stress with 3D echocardiography, after optimization of blood pressure if arterial hypertension is present), and if there is clear evidence of extensive valve calcification (as demonstrated by transesophageal echocardiography or computed tomography), particularly involving the commissures [63].

This flowchart outlines the step-by-step diagnostic approach to patients with suspected low-gradient aortic stenosis despite preserved left ventricular ejection fraction. The initial evaluation includes confirming the presence of a systolic murmur and left ventricular hypertrophy. In cases where the mean transvalvular gradient is <40 mmHg, aortic valve area, assessed using the continuity equation, guides further classification of aortic stenosis (mild, moderate, or <1 cm^2^ requiring flow assessment). If aortic valve area is <1.0 cm^2^, it is essential to evaluate flow conditions using stroke volume and flow rate to differentiate true severe stenosis from pseudo-severe forms due to measurement errors or low-flow states. Flow status is determined by stroke volume index and flow rate. Transesophageal echocardiography, calcium scoring with computed tomography, and dobutamine stress echocardiography can help clarify the severity and confirm the presence of valve reserve. In the presence of discordant findings or suspected myocardial disease (e.g., cardiac amyloidosis or hypertrophic cardiomyopathy), further evaluation with cardiac magnetic resonance and strain imaging may be required. The algorithm helps stratify patients into categories such as true severe stenosis, moderate stenosis, or low-flow states, guiding therapeutic decision making. AS: Aortic Stenosis; AVA: Aortic Valve Area; TVG: Transvalvular Gradient; 3D-TEE: Three-dimensional Transesophageal Echocardiography; CT: Computed Tomography; SV: Stroke Volume; FR: Flow Rate.

#### 3.3.2. B-Low-Flow Low-Gradient, Low Ejection Fraction with Discordant Aortic Valve Area (<1 cm^2^)

This phenotype includes severe AS with a low gradient (mean gradient < 40 mm Hg) due to low-flow conditions (Svi < 35 mL/m^2^), accompanied by a reduced LVEF (<50%) [64]. However, low-flow conditions may act as a confounding factor, leading to an underestimation of AVA calculation, a reduction in aortic valve opening, and a decrease in TVG. Consequently, in the presence of true low-flow conditions, AS metrics may be masked or misrepresented, leading to an indeterminate diagnosis of AS. An increase in flow (>20%) following the contractile recruitment of LV through dobutamine infusion may help unmask AS by increasing TVG without significant changes in AVA [65]. Conversely, an improvement in AVA without a significant increase in TVG following flow normalization supports the diagnosis of pseudo-AS. Planimetric AVA using TEE during dobutamine infusion may enhance diagnostic accuracy in distinguishing true AS from pseudo-AS, particularly in cases where an apparent rise in TVG is secondary to reduced blood pressure rather than fixed obstruction. A crucial step is the evaluation of flow conditions and their changes following left ventricular contractile recruitment. SV and FR are the most commonly used parameters for assessing flow conditions in AS. An SV of less than 35 mL/m^2^ or an FR of less than 200 mL/m^2^ defines a low-flow state, which limits the reliability of conventional AS assessment parameters. Therefore, identifying low-gradient AS in the context of low-flow conditions requires a reassessment of AS parameters after flow normalization. Dobutamine infusion is the most widely used test to assess left ventricular contractile reserve and aortic valve changes [66,67,68,69]. At low doses, dobutamine produces dissociation between chronotropic and contractile responses, making SV measurement a sensitive marker of flow changes. At higher doses, however, FR assessment is needed to refine the assessment of flow state improvement, preventing a misleading interpretation based solely on SVi. For a diagnostic purpose, an adequate flow reserve (increase in SV > 20%) should be achieved following dobutamine infusion. In the absence of adequate flow response, the projected valve at a normal FR can be estimated using the following formula: where D AVA and D Flow represent the absolute changes during DSE.

$${AVA}_{project}={AVA}_{rest}+\left(\left(\frac{∆AVA}{∆Flow}\right)*\left(250-{Flow}_{rest}\;\right)\right)$$

### 3.4. Normal Flow Low Gradient with Discordant Aortic Valve Area (<1 cm^2^)

This phenotype includes patients with AVA < 1 cm^2^, a mean gradient < 40 mmHg, but normal flow conditions assessed by SVi ≥ 35 mL/m^2^ and preserved LVEF (>50%) [70]. This patient subgroup presents a particular diagnostic challenge and requires careful evaluation to exclude measurement errors and distinguish false AS from significant AS [71,72,73,74]. Reduced aortic compliance and associated systolic hypertension may contribute to a substantial decrease in TVG, leading to the NF-LG AS phenotype [75]. The diagnosis and management of patients with NF-LG AS remain subjects of ongoing debate. A crucial point of discussion is the reliance on SVi measurement to determine flow status. In symptomatic patients, additional diagnostic tests are required to confirm AS severity and guide appropriate management. In particular, FR measurement in addition to SVi may be a useful parameter for refining the classification of a true low-flow state, especially in patients with reduced heart rate and/or a small body surface area. Exercise Echocardiography through SVi and FR increase may unmask significant TVG in patients with baseline normal flow low gradient AS. The use of TEE (or CT/CMR) may provide more accurate AVA measurements along with LVOT-CSA assessment for flow estimation. Additionally, CT-derived calcium scoring can help confirm severe AS in cases where the pressure gradient is reduced. Another source of debate concerns the AVA cut-point value of 1.0 cm^2^, as proposed in current guidelines for defining severe AS. This threshold does not correspond to a mean gradient of 40 mmHg but rather to a range of 30–35 mmHg. Consequently, some investigators have suggested lowering the severity cut-point for AVA from 1.0 to 0.8 cm^2^. However, multiple studies have demonstrated that an AVA threshold of 1.0 cm^2^ remains the most reliable predictor of mortality. Since a lower SVi is a strong predictor of outcomes both before and after AVR, patients with NF-LG AS could be considered at a less advanced stage of the disease, with potentially better survival compared to those with paradoxical LF-LG or high-gradient AS, provided that the measurement errors are excluded and normal flow conditions are categorized with concordant FR. However, several studies have reported conflicting outcomes after valve replacement in this population. These discrepancies underscore the substantial heterogeneity within this subgroup and reinforce the need for a nuanced, patient-specific treatment approach [76,77,78,79,80,81].

## 4. Trajectory of Transvalvular Aortic Gradient

The valvular substrate underlying AS typically begins with mild lesions and progresses toward severe calcification, resulting in an increasing obstructive burden that may remain clinically stable, develop significant symptoms, or acutely manifest clinical instability [82]. As the valve leaflets become progressively stiff, the restricted aortic valve opening leads to a parallel increase in TVG. Under normal flow conditions and with preserved LV function, TVG can serve as an accurate parameter to monitor the progression of AS severity. A systematic assessment of TVG may be useful, in the absence of high-flow states, to define the trajectory of AS severity up to its most advanced stage (extreme AS with peak TVG > 100 mm Hg, V > 5.5 m/sec). However, LV dysfunction and reduced flow conditions can lead to a decrease in TVG despite a worsening AS burden, potentially resulting in a misleading underestimation of disease severity. Progressive LV dysfunction and low-flow states may cause a reversal of previously elevated TVG values, culminating in an “amputated” AS stage characterized by severe AS with a non-significant TVG. Ideally, systematic monitoring of TVG in combination with flow parameters could help to more accurately define the specific trajectory of AS in individual patients, including the transition to the extreme stage and eventual low-gradient (“amputated”) AS. In this context, progressive valvular obstruction, coupled with variations in heart rate, left ventricular function, and vascular impedance, may lead to a reduction in the TVG despite flow conditions remaining within the normal range. Recognition of a progressive or regressive (declining) trajectory of TVG is paramount in the evaluation of individual patients. In the absence of systematic monitoring, an isolated TVG measurement must be interpreted within a comprehensive hemodynamic context to accurately define the stage of AS. Specifically, flow parameters (e.g., SVi, FR) and LV function indices (e.g., EF, global longitudinal strain [GLS]) are essential to determine whether the current TVG reflects a progressive, compensated phase or a maladaptive reduction from previously higher values due to impaired flow or LV dysfunction. For example, when observing a similar TVG value (e.g., a mean gradient of 40 mm Hg), the presence of reduced flow conditions may suggest a regressive trajectory from a previously higher gradient. In contrast, the same TVG under normal flow conditions would indicate a progressive trajectory of AS. A focused analysis of the LV myocardium burdened by valvular obstruction may help clinicians discern progressive or subtle declines in TVG across the varied presentations of AS [83,84,85,86,87,88,89,90,91].

## 5. A Comprehensive Approach to Multifaceted Transvalvular Gradient in Aortic Stenosis

**Figure 4:**
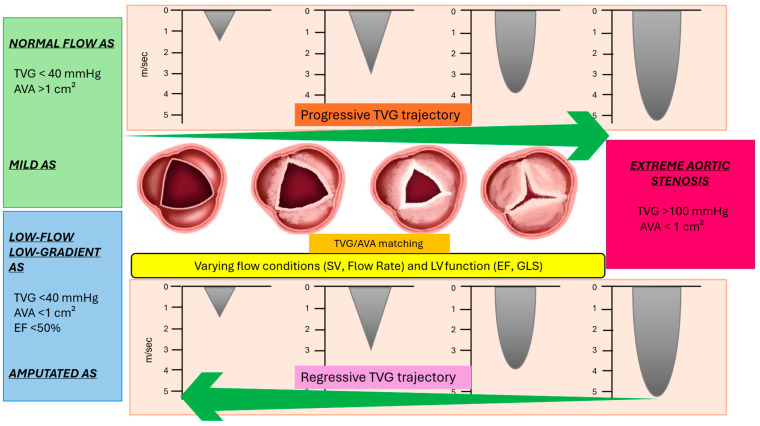
Hemodynamic and Anatomical Trajectory of Aortic Stenosis. Schematic representation of aortic stenosis (AS) progression based on transvalvular gradient (TVG), aortic valve area (AVA), and left ventricular (LV) function indices. The central row illustrates the progressive anatomical degeneration of the aortic valve from mild to extreme AS, accompanied by corresponding Doppler-derived increases in TVG. The top trajectory (green) shows a progressive rise in TVG with preserved flow, whereas the bottom trajectory (red) reflects a regressive pattern, characterized by a “backwards” declining TVG in the context of low-flow states, eventually leading to reduced ejection fraction (EF). The yellow band highlights the impact of varying flow conditions (stroke volume [SV], flow rate) and LV performance (EF, global longitudinal strain [GLS]) on the hemodynamic presentation of AS. Accurate grading requires appropriate matching between TVG and AVA.

Assessing AS is a complex task due to the multifaceted nature of TVG in the context of valvular obstruction. A comprehensive approach is therefore essential, incorporating a multiparametric analysis of TVG that includes AVA, flow dynamics, vascular impedance, and LV function indices. This integrated assessment is required to clarify the hemodynamic burden of valvular obstruction, identify worsening trajectories, and unmask concealed or underestimated TVG. Assessment of the TVG requires careful attention and the use of multiple echocardiographic windows to ensure the highest transvalvular velocity is captured. In particular, systematic use of the right parasternal window is crucial to detect the maximal TVG. Additionally, accurate measurement of LVOT velocity is essential to determine the applicability of the simplified Bernoulli equation in calculating TVG. Determination of the AVA using the continuity equation is the next step to support the diagnosis of severe AS. Following the identification of a significant TVG (mean TVG > 40 mm Hg), assessing flow conditions through SVi and FR is critical to contextualize the observed gradient. Excluding and correcting for high-flow states is necessary to avoid overestimation of TVG and misclassification of severe or extreme AS. In cases where a high TVG is discordant with a non-severe AVA (>1 cm^2^), planimetric assessment via TEE can refine the estimation of true AVA, particularly in patients with doming or bicuspid aortic valve morphology or in high flow conditions. If TEE confirms a discordance between TVG and AVA, the next step is to evaluate the contribution of pressure recovery, which may underlie the elevated TVG despite a non-severe AVA. Several factors may contribute to significant pressure recovery, such as a small ascending aorta or a centrally directed systolic transvalvular jet. The evaluation of the energy loss index (ELI) is a useful parameter to estimate the net hemodynamic burden on the left ventricle. An ELI < 0.6 cm^2^/m^2^ is considered a reliable prognostic indicator, even in the presence of a varying baseline TVG. In cases where a high TVG is concordant with the AVA, further assessment of LV hemodynamic burden, through indices such as EF and GLS, along with flow estimation, may help refine the TVG-trajectory of AS. In asymptomatic patients, a high TVG combined with a TVG-worsening trajectory, indicated by low flow or LV dysfunction, may be associated with an adverse prognosis. Moreover, the presence of concomitant conditions that reduce pressure recovery—such as a dilated ascending aorta, eccentric transvalvular jet, or dome-shaped stenosis—supports the notion of an increased LV energetic cost associated with a high TVG. This burden can be quantified by an energy loss index (ELI) < 0.6 cm^2^/m^2^, which reflects a more severe hemodynamic impact of TVG in the setting of comorbidity. Special attention is required during AVA measurement to minimize potential estimation errors, particularly in patients with distorted LVOT geometry. Additionally, indexing AVA to body surface area (BSA) is essential to avoid overestimating obstruction severity, especially in individuals of smaller stature. Figure 4 presents a model to identify severe AS, functioning as a clinical “road map.” This model integrates multiple diagnostic indices that account for the varying degrees of concordance between TVG and AVA values. By applying these principles, AS classification moves beyond a binary reliance on AVA and TVG, incorporating SV/FR and LV contractile function, including EF, to refine diagnostic accuracy. The severity of AS exists along a spectrum defined by the degree of agreement between TVG and AVA, with each end of the spectrum associated with distinct prognostic profiles. At one extreme, patients with markedly elevated TVG (peak velocity > 5.5 m/s) typically exhibit a concordant reduction in AVA, facing an unfavorable prognosis—even in the absence of symptoms. At the opposite end, concordance between a low TVG and an AVA > 1.0 cm^2^, combined with normal flow, is associated with a more favorable clinical course. Between these two poles, TVG may progressively increase while AVA declines; however, given the flow-dependent nature of both parameters, a wide array of TVG–AVA combinations may be observed. Consequently, adjustments for SV and vascular impedance are critical in identifying patients with significant AS that might remain undiagnosed under low-flow conditions, despite preserved LVEF. Furthermore, LV remodeling and the presence of arterial hypertension must be carefully considered, as they can contribute to underestimation and diagnostic inaccuracies. A comprehensive evaluation that integrates flow conditions and aortic vascular impedance offers a more nuanced understanding of AS severity, enabling a more accurate diagnosis and improved risk stratification with concordant obstruction findings. Exercise stress echocardiography (ESE) enhances the clinical assessment of AS by providing an integrated evaluation of the hemodynamic response to exercise. Unlike dobutamine stress echocardiography, ESE objectively defines functional status through the unmasking of exertional symptoms. Although flow and systemic vascular responses may modulate TVG and AVA, ESE can expose maladaptive hemodynamic patterns, such as exercise-induced hypotension or a rise in pulmonary pressure, thereby refining risk stratification in asymptomatic patients [92].

## 6. Challenges in Diagnosing Aortic Stenosis in Complex Clinical Contexts

The identification of AS based on TVG assessment can be particularly challenging in concomitant complex clinical contexts such as hypertrophic cardiomyopathy, cardiac amyloidosis, atrial fibrillation, ventricular dyssynchrony, and other coexisting cardiac pathologies.

### 6.1. Hypertrophic Cardiomyopathy (Personal Observation)

In hypertrophic cardiomyopathy (HCM), reliance on TVG measurements for the diagnosis of AS may be confounded by dynamic LVOT obstruction and abnormal loading conditions, potentially leading to both underestimation and overestimation of stenosis severity. Dynamic LVOT obstruction limits the applicability of the simplified Bernoulli equation for velocity-derived TVG estimation. Moreover, effective forward SV is often reduced due to the combined effects of obstruction and systolic anterior motion (SAM)-related mitral regurgitation, resulting in low-flow conditions and an apparent reduction in the calculated AVA (a phenomenon that may resemble high-gradient pseudo-aortic stenosis). Conversely, in patients with true AS and elevated afterload, LVOT obstruction and SAM-related mitral regurgitation may remain clinically silent until unmasked following aortic valve replacement. Flow assessment may be further hampered by technical inaccuracies of Doppler and volumetric methods, due to anatomical and rheological LVOT reshaping and unreliable LV volume estimation, respectively. In addition, the presence of SAM-related mitral regurgitation may shorten the ejection period, potentially preserving flow rate despite a low stroke volume. Transesophageal echocardiography may improve diagnostic accuracy in the setting of coexisting HCM and AS, particularly when early systolic valve opening followed by partial closure is observed, supporting a diagnosis of pseudo-stenosis. Dobutamine stress echocardiography may further enhance diagnostic distinction by restoring full aortic valve opening and unmasking dynamic LVOT obstruction, thus aiding in the recognition of pseudo-stenosis. Conversely, in the absence of AVA reserve, dobutamine may reveal true AS but fail to elicit latent LVOT obstruction. Finally, in some patients with HCM, progressive calcific AS may develop over time, gradually masking the pre-existing dynamic obstruction, thereby complicating both diagnosis and management.

### 6.2. Aortic Stenosis and Atrial Fibrillation

Atrial fibrillation (AF) can complicate the assessment of AS because beat-to-beat variability in diastolic filling alter SV, producing fluctuations in aortic jet velocity and TVG. Shorter preceding R-R interval yields lower SV and velocity reduced diastolic filling, whereas longer R–R interval increases both SV and diastolic filling, making severity assessment based on a single beat unreliable. Current guidelines recommend averaging multiple representative beats with relatively uniform R-R intervals. It is also essential to select representative cardiac cycles (avoiding higher R-R interval and post-extrasystolic beats, which are associated with high output due to a compensatory pause and a transient fall in arterial resistance). In clinical practice, it may be helpful to control the patient’s heart rate to 60–70 bpm, when feasible, and selecting cycles with similar R-R intervals for measurement can improve reliability. Despite these precautions, Doppler-derived gradient often underestimates AS severity in the setting of AF [93,94,95,96]. In selected scenarios, particularly when AF is newly diagnosed in a patient with suspected AS, restoring sinus rhythm, via beta-blockade or cardioversion when appropriate, may permit a more accurate assessment of AS severity. Notably, because TEE allows beat-to-beat evaluation, assessing it during longer R-R intervals or following a premature ventricular contraction may reveal AVA reserve, helping to rule out severe AS in cases of low-flow, low-gradient states. CT Calcium Score is explicitly recommended in Guidelines as a marker of AS severity when echocardiographic measurements are discordant or difficult to interpret using Calcium score ≥ 2000 (men) or ≥1250 (women). Exercise or dobutamine stress echocardiography may also help unmask true AS severity in low-flow situations by increasing stroke volume. In routine practice, a dobutamine stress echo is recommended for patients with low-flow, low-gradient AS and reduced EF to distinguish true severe AS from pseudo-severe disease. In AF patients, however, dobutamine must be used cautiously, as it increases the risk of tachyarrhythmias during catecholamine infusion.

## 7. Perspectives

Despite its broad diagnostic and therapeutic implications, TVG requires an integrated interpretative approach, tailored to the underlying pathophysiological substrate influencing the measured value. Further advances in understanding are needed to gain deeper insight into the complex burden of AS.

### 7.1. The Concept of Aortic Valvular Reserve

The echocardiographic assessment of AS severity is traditionally based on parameters of valvular obstruction measured at rest. However, resting indices do not always correlate with the clinical presentation [8]. A substantial proportion of patients remain asymptomatic despite being classified as having severe AS at rest [97]. Planimetric evaluation of AVA with TEE, both at baseline and during dobutamine stress, enables assessment of aortic valvular reserve. This term refers to the capacity of the valve to increase its effective orifice area in response to stress, whether pharmacological or physiological exertion. The concept extends beyond static measures of stenosis severity and provides insight into the dynamic functional status of the valve and its interaction with the left ventricle. Recognition and evaluation of valve reserve may be particularly relevant in borderline cases or when symptoms and resting hemodynamic indices are discordant. From a pathophysiological standpoint, valve reserve is modulated by both LV contractile reserve and the residual elasticity of the cusps. Extensive calcification, especially at the commissures, is a major limiting factor of valve opening reserve and may indicate a worse prognosis. In patients undergoing valvuloplasty, restoration of cusp elasticity under pharmacological stress (e.g., sodium nitroprusside) has been associated with clinical improvement, even when resting AVA remained critically low [98]. Thus, valve reserve testing represents a potentially valuable tool for prognostic stratification in asymptomatic patients otherwise categorized as having severe AS at rest. No universally accepted threshold exists to define “preserved” versus “reduced” reserve, and stress-testing protocols should be individualized based on patient tolerance and clinical goals. Valve reserve may be a one component of a complex diagnostic puzzle and must be considered alongside LV function, symptom burden, and comorbidity conditions.

### 7.2. Potential Clinical Implications of Aortic Valve Reserve

**Figure 5:**
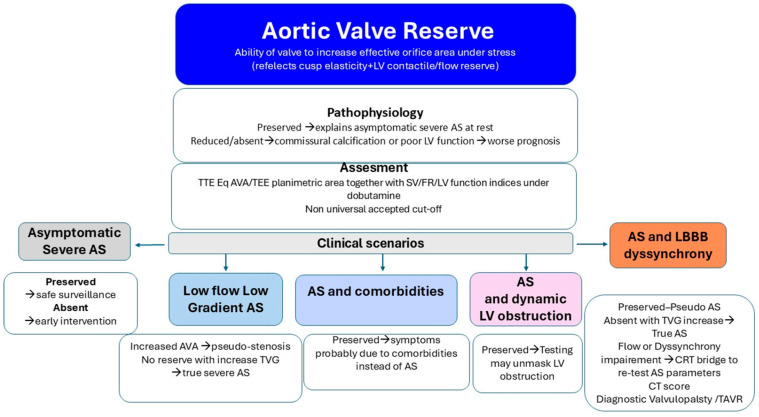
Valve reserve testing to refine risk stratification, distinguish true from pseudo-severe stenosis, and guide timing of intervention.

The presence or absence of aortic valve reserve may influence AS management in multiple ways. (Figure 5).

**Figure 6:**
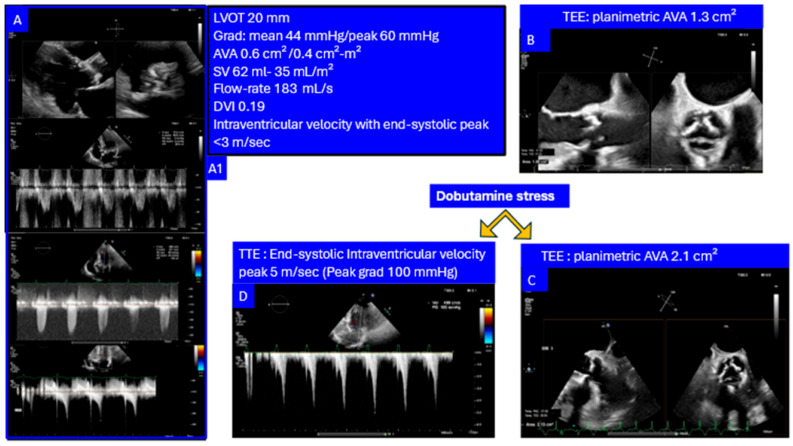
Echocardiographic evaluation of high TVG suggesting severe aortic stenosis with concomitant inducible dynamic intraventricular obstruction Dobutamine stress induced a marked rise in end-systolic intraventricular velocity (5 m/s, peak gradient 100 mmHg), with TEE planimetry confirming functional valve area reserve. (**A**): baseline TT examination (see (**A1**) box). (**B**): Baseline TEE. Dobutamine stress test (**C**): TEE showing increase in AVA; (**D**): Doppler TT showing an impressive intraventricular gradient).

‑ *Asymptomatic Severe AS.* Emerging data suggest that early valve replacement in patients with asymptomatic severe aortic stenosis may improve outcomes compared with a surveillance strategy, which cannot reliably predict the onset of acute valve syndromes [99]. In patients with severe AS parameters at rest, preserved valve reserve may justify watchful waiting with close follow-up. Conversely, absent reserve can support earlier intervention despite a lack of overt symptoms.
‑ *Low-Flow, Low-Gradient AS* vs. *Pseudo-Stenosis.* Recruitment of LV contractile reserve during inotropic stimulation may enhance valve opening in patients with reduced baseline excursion, supporting a diagnosis of pseudo-stenosis. In contrast, absent valve reserve with increased TVG indicates true (“masked”) severe AS requiring valve replacement.
‑ *Symptoms in Severe AS with Comorbidity*. Several studies have shown a high rate of readmission for heart failure or a lack of symptomatic improvement following TAVI, suggesting a potentially underrecognized burden of comorbidities [100,101,102]. Valve reserve assessment acknowledges that static measurements may underestimate the hemodynamic impact of obstruction or fail to capture the valve’s latent adaptability. Demonstration of preserved valve reserve suggests that comorbid conditions may be the primary drivers of symptoms, highlighting the importance of comprehensive evaluation in complex and often challenging clinical settings.
‑ *AS with Intraventricular Dynamic Obstruction*. Increased afterload from AS can obscure dynamic intraventricular gradients, which may become apparent after valve replacement. Conversely, preserved valve reserve during dobutamine stress may unmask the dynamic component before intervention supporting a diagnosis of non-significant AS with inducible dominant dynamic LV obstruction as a main cause of symptoms (Figure 6).

*-AS with LV Dyssynchrony.* In patients with left bundle branch block (LBBB), LV dyssynchrony may reduce flow, leading to underestimation of AS severity [103]. Planimetric AVA measurement with TEE can identify pseudo-stenosis when the opening area exceeds 1 cm^2^ and TVG is less than 40 mmHg. Conversely, when AVA is <1 cm^2^ and TVG < 40 mm Hg in conditions of reduced flow, additional tools such as CT calcium scoring or TEE demonstration of commissural fusion are supportive signs of true AS. Dobutamine stress may elicit TVG recruiting flow but can worsen dyssynchrony at higher heart rates inducing further flow impairment potentially obscuring the AS diagnosis [104]. Cardiac resynchronization therapy (CRT) may improve flow conditions and diagnostic accuracy. Alternatively, discordant findings (low TVG, small AVA, heavy commissural calcification, absent contractile reserve) may justify “diagnostic valvuloplasty” or direct referral for transcatheter aortic valve replacement.

### 7.3. Refinement of Flow Assessment

Flow assessment is a crucial aspect of the diagnostic work-up and management of AS. However, obtaining accurate flow measurements can be challenging due to operator-dependent variability, particularly in cases with distorted LVOT geometry. The recent introduction of automated systems for dynamic left ventricular assessment may help overcome the limitations of conventional methods in evaluating flow status. Automated flow assessment can improve the accuracy of detecting dynamic flow changes during stress testing, including dobutamine and exercise echocardiography, thereby refining the diagnostic evaluation of AS [105,106].

### 7.4. Fluid Dynamics: A New Exploratory Frontier in Aortic Stenosis

Intraventricular fluid dynamics through vortex formation plays a critical role in maintaining efficient cardiac function [107,108,109]. This coordinated flow pattern reduces energy dissipation, minimizes wall stress, and promotes effective SV with minimal mechanical load. Disruptions of these vortex patterns may promote adverse LV remodeling and progression toward heart failure [107]. Analyzing LV vortex flow patterns in the context of cardiac remodeling, advanced imaging techniques such as 4D Flow MRI, vector flow mapping, and contrast echocardiography indicate that vortex characteristics (e.g., kinetic energy, vorticity, and flow organization) reflect subtle mechanical changes in the heart even before conventional markers (such as ejection fraction) show abnormalities. In the setting of AS, fluid dynamic impairment may enhance the understanding of the hemodynamic burden caused by valvular obstruction. In patients with AS, the progressive narrowing of the aortic valve orifice increases afterload and may alter flow trajectories within the LV. As a result, vortex patterns may become disorganized, asymmetric, or diminished in intensity. This loss of coherent vortex structure may contribute to inefficient ejection, increased turbulence, and abnormal redistribution of intraventricular pressure gradients. Over time, these changes can accelerate adverse LV remodeling, promote diastolic dysfunction, and contribute to the transition toward heart failure (even in the presence of preserved ejection fraction). This could alert clinicians to the need for closer surveillance, even in the absence of symptoms. Thus, the integration of advanced flow assessment into the diagnostic algorithm for AS may open a promising frontier in precision cardiology. The analysis of intraventricular flow, particularly vortex behavior, may enhance risk stratification in AS by identifying patients at higher risk for deterioration, despite preserved conventional metrics.

In addition, analysis of fluid dynamics in the ascending aorta may further elucidate the hemodynamic burden of AS [108]. In particular, the vorticity of aortic flow may be altered by the eccentricity of transvalvular flow in AS, contributing to energy dissipation. Clinical assessment of aortic flow hemodynamics using advanced multimodality cardiovascular imaging (particularly 4D-flow CMR) may refine AS phenotyping and risk stratification, thereby supporting timely, targeted planning of aortic valve interventions [109,110,111,112,113]. Despite promising results, more standardized methodologies and larger multicenter trials are still needed to validate these parameters as routine clinical tools.

## 8. Conclusions

Understanding the multifaceted nature of TVG is crucial to refine the diagnosis and management of AS. Assessing flow conditions enables TVG phenotyping, helping to reconcile discrepancies with the AVA measurements. Dynamic assessment strategies further refine the interpretation of standard parameters, enhancing their applicability across a range of clinical scenarios, from routine evaluations to complex cases. Furthermore, an integrative analysis of valvular obstruction burden, flow dynamics, and LV function, including advanced fluid dynamics, can provide deeper insight into both the current state and the trajectories of TVG, ultimately contributing to more tailored and effective AS management.

A transvalvular gradient (TVG) greater than 100 mmHg or a velocity exceeding 5.5 m/s defines very severe aortic stenosis and is associated with a significantly increased short-term risk of adverse events, irrespective of valve area.

The same TVG may impose different energetic burdens on the left ventricle depending on factors that influence pressure recovery distal to the stenosis, such as aortic dimensions, jet eccentricity, and stenotic geometry.

Normalizing the gradient for flow conditions and vascular impedance enables a more accurate assessment of stenosis severity and provides improved prognostic stratification.

A comprehensive evaluation of left ventricular function and flow state may clarify whether the trajectory of the transvalvular gradient is progressive or regressive (declining), supporting either a “watchful waiting” or interventional strategy in asymptomatic patients.

Baseline obstruction can be further stratified using stress echocardiography, which may unmask a maladaptive exercise response or reveal aortic stenosis with a blunted transvalvular gradient.

Aortic valvular reserve assessed with inotropic or vasodilator agents, may reflect a hemodynamic adaptation mechanism to exertion and assist in the evaluation of aortic stenosis in complex clinical contexts (e.g., cardiac co-pathologies, comorbidities).

## Data Availability

No new data were created or analyzed in this study.
